# Multifaceted transcriptional regulatory pathways keep plant immune responses under control

**DOI:** 10.1093/plphys/kiag151

**Published:** 2026-03-18

**Authors:** Alyssa Kearly

**Affiliations:** Assistant Features Editor, Plant Physiology, American Society of Plant Biologists, Rockville, MD, United States; Boyce Thompson Institute, Cornell University, Ithaca, NY 14853, United States

To defend against bacteria, fungi, viruses, and parasites, plants rely on a surveillance system of receptor proteins to detect pathogens and mount immune responses. One such class of receptor proteins, nucleotide-binding leucine-rich repeat receptors (NLRs), detects pathogen effectors inside host cells and initiates effector-triggered immunity (ETI). Activation of NLRs leads to broad changes in gene expression that include upregulation of defense genes and repression of genes associated with growth. Mounting an immune response, therefore, comes at the cost of growth and development. Thus, the expression of NLRs must be tightly controlled under normal conditions to prevent inappropriate activation of immune responses.

Plant cells employ a variety of mechanisms to precisely coordinate where a gene is expressed and at what level. Local chromatin architecture, transcription factor binding, histone composition, and histone post-translational modification work combinatorially to achieve the gene expression patterns that define cell identities. Then, in response to cellular stimuli, these aspects can all be overhauled to mediate the necessary gene expression changes. The transcriptional regulation of the NLR *SUPRESSOR OF NPR1, CONSTITUTIVE 1* (*SNC1*) has been extensively studied and is known to be impacted by the binding of transcription factors to SNC1 regulatory elements and histone post-translational modifications throughout the *SNC1* locus (Fig. 1). Yang et al. (2026) add additional depth to our understanding of this regulation in their recent work published in *Plant Physiology*, in which they explored the roles of the histone chaperones *CALCINEURIN BINDING PROTEIN 1* (*CABIN1*) and HISTONE REGULATOR A (HIRA) in the transcriptional regulation of immune responses.

**Figure 1:**
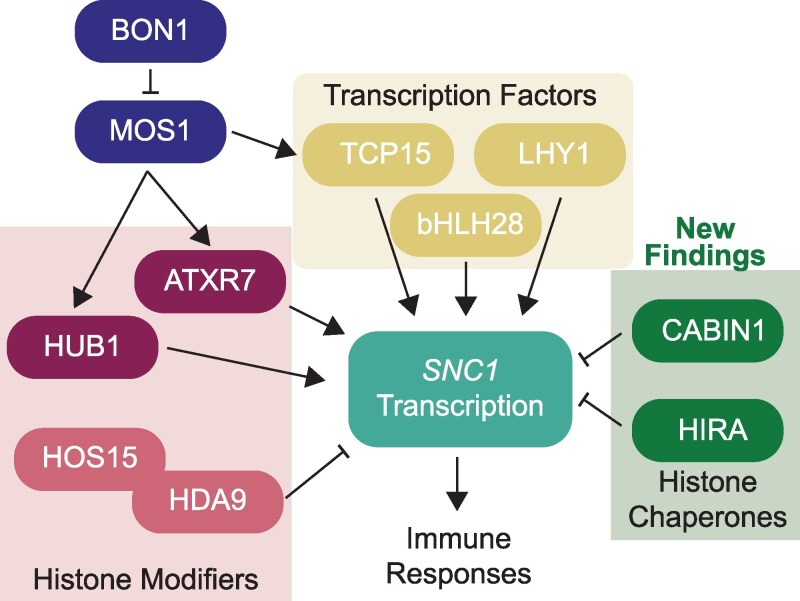
The complex transcriptional regulation of *SNC1*. Expression of NLRs like *SNC1* must be tightly controlled to prevent the inappropriate activation of immune responses. Previous studies have shown that transcription factors and histone modifiers impact transcription of the *SNC1* gene. In terms of histone modifiers, the methyltransferase TRITHORAX-RELATED7 (ATXR7) and the E3 ubiquitin ligase HISTONE MONOUBIQUITINATION1 (HUB1) promote *SNC1* expression (Xia et al. 2013; Zou et al. 2014), while HIGH EXPRESSION OF OSMOTICALLY RESPONSIVE GENES 15 (HOS15) works with HISTONE DEACETYLASE 9 (HDA9) to repress *SNC1* (Yang et al. 2020). Transcription factors TEOSINTE BRANCHED 1 (TCP15), LATE ELONGATED HYPOCOTYL 1 (LHY1), and BASIC HELIX-LOOP-HELIX 28 (bHLH28) activate *SNC1* expression (Zhang et al. 2018; Yu et al. 2023). MOS1 cooperates with HUB1, ATXR7, and TCP15, but its activity is limited by BON1 under normal conditions. In the current study, the researchers uncover 2 histone chaperones, CABIN1 and HIRA, that negatively regulate *SNC1* expression independent of the BON1-MOS1 axis.

Previous studies showed that SNC1 expression is controlled by the proteins BONSAI 1 (BON1) and MODIFER OF *snc1* (MOS1; Fig. 1). BON1 is a membrane-localized calcium sensor that, under basal conditions, activates efflux systems to maintain low calcium levels in the cytosol (Li et al. 2025). *BON1* loss-of-function mutation results in the upregulation of *SNC1*, mediated by MOS1 and its cooperative binding with histone modifiers and transcription factors at the *SNC1* locus (Xia et al. 2013; Zou et al. 2014; Zhang et al. 2018). Consequently, *bon1* single mutants display severe growth defects, basal immune activation, and enhanced resistance to pathogens, reflective of an autoimmune phenotype (Yang and Hua 2004). Importantly, this phenotype is dependent on MOS1 function, and so the effects of BON1 loss are ameliorated in *bon1 mos1* double mutants (Bao et al. 2014). To explore *SNC1* regulation beyond this BON1-MOS1 axis, Yang et al. (2026) performed a suppressor screen on *bon1 mos1* double mutants, searching for mutations that reintroduced the autoimmune phenotype of the *bon1* single mutant. One such mutation, dubbed *smo4*, conferred *bon1*-like growth defects and increased resistance to the bacterial pathogen *Pseudomonas syringae* alongside increased basal expression of both *SNC1* and defense marker *PATHOGENESIS-RELATED PROTEIN 1* (*PR1*). The mutation was mapped back to the gene encoding histone chaperone CABIN1.

Two independent *cabin1* single mutants displayed attenuated autoimmune phenotypes compared with *bon1* mutants. Morphologically, *cabin1-2* and *cabin1-3* plants were indistinguishable from wild-type (WT) plants aside from a slight decrease in fresh weight, and they were less resistant to *Pseudomonas* infection compared with *bon1* plants, though still more resistant than WT. Similarly, expression of *SNC1* and *PR1* was upregulated to a lesser extent in *cabin1* plants compared with *bon1*. Transcriptome-wide analysis of *cabin1-3* and WT plants under normal conditions indicated basal immune activation in the mutants, with immune- and stress-associated genes enriched in their set of upregulated genes, implicating CABIN1 in the negative regulation of immune responses in the absence of pathogens. Further genetic studies suggested that CABIN1-driven autoimmunity depends on the central salicylic acid signaling regulator *PHYTOALEXIN DEFICIENT 4* (*PAD4*) and, interestingly, on *SNC1* itself.

CABIN1 homologs in other species have been shown to impact transcription in 2 ways: (1) through calcium-dependent interactions with DNA-bound transcription factors and subsequent recruitment of repressive histone modifiers (Youn and Liu 2000; Jang et al. 2007); and (2) in complex with HIRA and UBINUCLEIN (UBN), depositing the active transcription-associated histone variant H3.3 in regions of euchromatin (Rai et al. 2011; Szenker et al. 2011). Therefore, Yang et al. (2026) sought to determine if Arabidopsis CABIN1 regulates *SNC1* expression through these strategies. First, genome-wide analysis of CABIN1 binding revealed its enrichment in promoter-proximal regions, including at the *SNC1* transcription start site, suggesting that CABIN1 may regulate *SNC1* expression through direct binding of its promoter. CABIN1 was also determined to interact with HIRA and could therefore be influencing histone composition to modulate *SNC1* expression as well.

The researchers then further explored how HIRA fits within the pathways regulating *SNC1* expression using additional genetic studies. The loss of *HIRA* generally imparted phenotypes similar to those seen with the loss of *CABIN1*, in both the WT and the *bon1 mos1* backgrounds, with intermediate levels of pathogen resistance and upregulation of *SCN1* and *PR1* compared with WT and *bon1* single mutants. In the *bon1* background, either *cabin1* or *hira* mutation synergistically worsened the autoimmunity-associated growth defects, suggesting that CABIN1, HIRA, and BON1 are negative regulators of immune responses that function, at least partially, in independent pathways (Fig. 1).

Because both CABIN1 and HIRA function as histone chaperones, the researchers next sought to explore their impact on levels of H3. Similar to previous reports in *hira* mutant seeds (Layat et al. 2021), *hira* and *cabin1* seedlings at 1 and 2 weeks both had reduced H3 levels compared with WT seedlings, though H3 levels rivaled those of WT by 3 weeks. Targeted examination of the H3.3 variant at the *SNC1* locus did not yield conclusive results, perhaps due to technical limitations of the experiments (e.g., use of protoplasts in lieu of whole seedlings or GFP fusion potentially impacting H3.3 incorporation into histones). However, it is alternatively possible that CABIN1 and HIRA do not regulate *SNC1* expression through their roles in shaping histone composition.

The work described by Yang et al. (2026) thus highlights the incredible nuance involved in the regulation of NLR proteins and immune responses more generally. Multiple regulatory pathways participate in fine-tuning the expression of the NLR *SNC1* (Fig. 1), ensuring that its expression is kept low under normal conditions to prevent basal immune activation that negatively impacts growth. Interesting questions remain regarding the presence of CABIN1 at the *SNC1* promoter. As CABIN1 lacks a DNA binding domain, further study may reveal interactions with transcription factors that influence the recruitment of histone modifiers to modulate transcription of *SNC1*, similar to findings in mammals. The exact mechanisms by which HIRA and CABIN1 regulate gene expression remain elusive, as do the details of how the various *SNC1* regulatory pathways interact. However, Yang et al. (2026) have set the stage for future efforts to fully understand the complex regulation of the intricate gene expression patterns underlying plant immunity.

## Recent related articles in *Plant Physiology*:

- Candela-Ferre et al. (2024) reviewed how DNA modifications, histone composition, and histone modifications impact chromatin accessibility and, by extension, gene expression.

## Data Availability

No new data was generated for this publication.
